# A self-management support intervention for patients with atrial fibrillation: a randomized controlled pilot trial

**DOI:** 10.1186/s40814-020-00624-y

**Published:** 2020-06-18

**Authors:** Stefan Jobst, Lynn Leppla, Stefan Köberich

**Affiliations:** 1 Faculty of Medicine, Institute of Nursing Science, University of Freiburg, Fehrenbachallee 8, D-79106 Freiburg, Germany; 2 Institute of Nursing Science, University of Basel, Bernoullistrasse 28, CH-4056 Basel, Switzerland; 3 Pflegedirektion, Heart Center University of Freiburg, Hugstetter Straße 55, D-79106 Freiburg, Germany

**Keywords:** Atrial fibrillation, Pulse palpation, Pulse self-palpation, Self-management, Self-monitoring, Symptom management, Nurse-led intervention, Pilot trial

## Abstract

**Background:**

Atrial fibrillation (AF) is the most common arrhythmia worldwide. Despite effective treatment, it is characterized by frequent recurrences. Optimal therapeutic management of AF requires active participation and self-management from patients. Two major components of self-management are self-monitoring and sign-and-symptom management. Pulse self-palpation (PSP) is a method of self-monitoring; however, not all AF patients are capable of successfully performing PSP. Due to a lack of interventions on this topic, a nurse-led intervention for patients with AF (PSPAF intervention) was developed to foster self-monitoring and to enhance self-management through PSP. The purpose of this pilot study was to test the acceptability, feasibility, and potential effects of this intervention on the capability of patients’ PSP and sign-and-symptom management. Moreover, we aimed at gathering data on the feasibility of applied research methods to aid in the design of future studies.

**Methods:**

The pilot trial involved 20 adult patients with AF, randomized to an intervention or usual care group. At baseline and during a home visit 3–5 weeks later, we collected data using questionnaires, checklists, field notes, a mobile ECG device, and a diary. Acceptability and feasibility measures were validated through predefined cut-off points. Effect size estimates were expressed as relative risks (RR) and the number needed to treat (NNT).

**Results:**

The PSPAF intervention seemed feasible, but only partly acceptable. There were limitations in terms of potential effectiveness, suitability, addressing participants’ willingness to implement its content in daily life, and adherence. Estimations of effect sizes suggest a large effect of the intervention on patients’ PSP capability (RR = 6.0; 95% CI = [0.83, 43.3]; NNT = 2.4), but almost no effect on sign-and-symptom management (RR = 1.5; 95% CI = [0.7, 3.1]; NNT = 4.0). The feasibility of applied research methods showed minor limitations on recruitment and participant burden.

**Conclusions:**

Despite some limitations, the intervention seemed to be applicable and promising. Taking into account the suggestions and amendments we have made, we recommend conducting a full-scale trial to examine the efficacy of the PSPAF intervention.

**Trial registration:**

This pilot study was registered in the German Clinical Trials Register at September 4, 2017 (Main ID: DRKS00012808).

## Background

Atrial fibrillation (AF) is the most common arrhythmia worldwide [1, 2] with an estimated 33.5 million people suffering from this illness [3]. The incidence and prevalence of AF increase, especially in the older population [3]. AF is a chronic condition associated with an increased risk of mortality [4], morbidity [5], as well as cognitive decline and dementia [6]. It is one of the major causes of stroke [5], impairs patients’ quality of life [7], and can cause depression [8] in affected patients. In addition, AF increases the economic burden of health care systems [9] due to increased absences from work, lower productivity [10], and higher utilization of health care services [9, 11–13].

The primary goals of AF management include stroke prevention, as well as heart rate and/or rhythm control [5]. The treatment of AF is complex [14] and challenging to manage in practice [15]. Furthermore, AF is characterized by frequent recurrences [16] with rates ranging from 22 to 83%, depending on therapy, time, and study [17–22]. In order to achieve optimal therapeutic management of AF and minimize the risk of complications, active patient participation is essential [23], as well as emphasizing the importance of patients’ self-management [24].

For the purpose of this study, we defined self-management as an “individual’s ability, in conjunction with family, community and the appropriate health care professionals, to successfully manage the symptoms, treatment, physical, psychosocial, cultural and spiritual consequences and inherent lifestyle changes required for living with a long-term chronic disease” ([24], p. 1145). Effective self-management of chronic conditions encompasses cognitive decisions to maintain physiologic stability as well as the recognition and response to symptoms [25]. In addition, self-management is associated with positive outcomes for patients and health care systems [25–29]. According to Richard and Shea [25], the concept of self-management is seen as an illness-related process embedded in the more broader concept of self-care. This, in turn, is associated with promoting and maintaining health.

Incorporated in the domain of self-management are the concepts of self-monitoring and symptom management. Self-monitoring is defined as the “awareness of symptoms or bodily sensations that is enhanced through periodic measurements, recordings and observations to provide information for improved self-management” ([30], p.343). For chronically ill patients, the recognition of symptoms marks the beginning of the decision-making process to which actions need to follow. Symptom management, when performed by patients, is seen as an element of self-monitoring and self-management [25]. Its aim is the delay and prevention of negative outcomes [31]. Besides symptoms as a subjective experience [31], signs are objective indications of a disease [32] and are considered to be important for recognizing problems [31]. The effective management of signs and symptoms is a process that starts with detection and interpretation, then leads to the selection of a strategy, and ends with an evaluation of the chosen strategy [33]. The manifestation and awareness of AF symptoms (such as lethargy, palpitations, dyspnea, chest tightness, sleeping difficulties, and psychosocial distress [5]) can vary greatly from symptom-free to a massive impairment of daily life [34–36]. AF recurrences can frequently be asymptomatic, and while patient reported symptoms are considered to be an inaccurate estimation [37], an irregular pulse, regarded as a clinical manifestation of or sign for AF, can be a measurable indicator of the disease or recurrence [38, 39] that can be detected through pulse palpation. In current guidelines, pulse palpitation is only recommended to health care providers as a method for screening individuals above the age of 65 [5] or as an assessment method for patients with specific symptoms [40]. Patient involvement in terms of pulse self-palpation (PSP) has not yet been explicitly considered in any guidelines.

PSP means feeling one’s own pulse wave with fingertips without the help of technical devices to determine the rate, rhythm, and quality of the pulse [41]. PSP is a simple, ubiquitous, non-invasive [42], cheap [43], useful, and safe [44] method to identify an irregular pulse. In studies, pulse palpation to assess heart rhythm has shown a sensitivity of 76%, a specificity of 86% when performed by the general population [45], and a rate-dependent accuracy of 82–92% when performed by the elderly [46]. A PSP by stroke patients unveiled a small number of false-positive measurements and achieved a sensitivity of 54% and a specificity of 96% [42].

Unfortunately, a limited number of AF-patients know that AF can be detected by regular pulse palpation [47], and even fewer are capable of performing PSP or in fact do so [23, 48]. In order to fill this gap and to promote patient involvement, it is recommended to educate patients with AF about PSP [49, 50].

Studies comprising PSP interventions focused on screening for AF and only included participants without AF [38, 42, 44, 45, 51, 52], but showed improvements in technique and capability of PSP, with rates ranging from 69 [44] up to 100% [52] in the intervention groups. These results suggest that inexperienced individuals can successfully learn PSP without negative outcomes. Interventions especially designed for AF patients focusing on self-management could not be identified in the published literature. For this reason, the “*P*ulse *S*elf-*P*alpation for patients with *A*trial *F*ibrillation (PSPAF)” intervention has been developed to foster self-monitoring and sign-and-symptom management, and thus to enhance self-management.

## Aims

The aim of this pilot trial was to test the PSPAF intervention and to gather data towards designing a future trial to test the efficacy of the intervention. Specific aims were (1) to assess the acceptability and feasibility of the intervention, (2) to assess the feasibility of research methods and the perceived burden of study participation, and (3) to estimate a potential effect of the intervention on the capability to perform PSP, and on sign-and-symptom management.

## Methods

Reporting on this pilot trial was guided by the recommendations of the CONSORT statement extension to randomized pilot and feasibility trials [53] (Reporting checklist: see Additional file 1).

### Design and setting

This single-center, single-blind randomized controlled pilot trial was conducted on a ward with a focus on heart rhythm diseases at an academic tertiary medical heart center in Germany.

### Participants, recruitment, and randomization

A sample of *N* = 20 participants (*n* = 10 per group) was targeted. This sample size was based on suggestions in the literature [54–56]. No formal sample size calculation was conducted.

Patients were included if they (1) were hospitalized, (2) had a diagnosis of paroxysmal or persistent AF, (3) were at least 35 years of age, (4) lived within 30 min by car from the health care center, and (5) were able to read, write, and understand German. Patients with (1) a cognitive (e.g., dementia) or (2) a physical impairment of both hands (e.g., peripheral polyneuropathy), (3) a third-degree heart block or condition after AV nodal ablation and an implanted pacemaker, (4) a life expectancy of less than 2 months according to the physician, or (5) individuals who had already taken part in any comparable education program, were excluded.

Screening of potential participants via electronic health records was carried out by the study coordinator. Eligible participants were informed about the trial both verbally and by written information. Patients who provided informed consent were randomized to either the intervention group (IG) or the usual care group (UCG) via sealed, opaque, and consecutively numbered envelopes containing paper cards with the group allocation. Group allocation was randomized by means of a computer-generated blocked randomization procedure with possible block sizes of 2, 4, 6, or 8 that were also randomly specified [57]. The confidential preparation and arrangement of envelopes was undertaken by a person not involved in any stages of this trial.

### Intervention

The nurse-led PSPAF intervention is a behavioral intervention at an individual level for one or two recipients, which can be referred to as “complex” due to several interacting components [58]. The intervention was developed by a group of experienced nurses and advisory cardiologists following the principles of action research [59] and was based on the four sources of evidence [60]: (1) empirical evidence, (2) clinical experience (involving nurses, physicians, and other health experts), (3) contextual factors, and (4) patient preferences (field testing), resulting in the creation of an intervention manual. Formal guidance of intervention development was retrieved from the work of Sidani and Braden [61].

The PSPAF intervention consisted of five consecutive components whose contents were presented orally in a face-to-face session by a trained registered nurse (study coordinator) using interactive teaching methods and written materials. Four main topics were addressed: (1) background information on the disease, pulse, and pulse measurement; (2) learning the technique of PSP; (3) determining heart rate and heart rhythm; and (4) interpretation of values and possible actions. To illustrate the mechanisms of the intervention, its active ingredients were classified as intervention functions [62] with a set of corresponding behavioral change techniques [63]. A detailed description of the PSPAF intervention is provided in Table 1.

**Table 1:** Consecutive components and content of the PSPAF intervention with corresponding intervention functions and behavioral change techniques

Components	Function	BCT	Content in PSPAF
1) Information	Education	Information about health consequences	Oral information about the pulse as a clinical sign, physiological and pathophysiological values, and the significance of measurement of one’s own pulse in terms of AF
2) Technique of a PSP	Education	Instruction on how to perform a behavior	Explanation of the procedure of a PSP
	Training	Demonstration of the behavior	Demonstration and joint exercise of the procedure of a PSP
3) Determination of heart rate and heart rhythm	Training	Instruction on how to perform a behavior	Elucidations and examples on how to determine heart rate and heart rhythm
	Training	Demonstration of the behavior	Demonstration and joint completion of how to determine heart rate and heart rhythm
4) Interpretation, action, and motivation	Training	Instruction on how to perform a behavior	Explanations on the interpretation of values
	Enablement	Action planning	Explanations of what actions to take with different heart rates and rhythm constellations
	Training	Behavioral practice/rehearsal	Stand-alone repetition of a complete PSP procedure (technique, determination of heart rate and heart rhythm, interpretation of values)
	Training	Habit formation	Prompt to perform a PSP at least twice a day: in the morning after breakfast and in the evening after dinner
	Training	Behavioral practice/rehearsal	Emphasis on and motivation to practice PSP more than two times/day in the first days after intervention
	Enablement	Verbal persuasion about capability	Verbal positive reinforcement of participants PSP capability
5) Delivery of supplementary material	Education	Information about health consequences	A fact sheet containing key information, illustrations, and explanation of the PSP process, with recommendations regarding sign and symptom management in the form of an algorithm displayed in a flowchart
	Education	Self-monitoring of behavior	Provision and explanation of a pulse diary where the values of heart rate and heart rhythm can be noted

*BCT* behavioral change techniques, *PSP* pulse self-palpation, *PSPAF* pulse self-palpation for patients with atrial fibrillation-intervention

### Variables and measurement

Variables and corresponding instruments are listed in detail in Table 2. All instruments were developed specifically for this study, based on literature or expert opinion. Therefore, no external evidence exists on their validity and reliability. In a cognitive pretest with nine individuals (three AF patients, five healthy individuals, one cardiac nurse), written study information, questionnaires, and the pulse diary were tested for clarity, readability, and comprehensibility using verbal probing and think-aloud interviews [64]. Vague or ambiguous items and formulations were revised. Regarding applicability and usability, field-note forms and checklists were explained to study assistants and discussed in advance with one assistant. The checklist assessing the capability of performing PSP and the vignettes assessing sign-and-symptom management were discussed with two expert nurses and one physician to gauge the accuracy of measuring the respective concepts.

**Table 2:** Description of outcome variables, instruments, and cut-off points

Outcome variable	Label of instrument	Description of instrument *Type of instrument* Sub-concept/-variable Items/scales/assessment	Time of measurement; group	Scale calculation/cut-off points
Demographic and clinical characteristics of participants	Q1	*Self-reported questionnaire* Demographic data (4 items) ▪ Age (in years) ▪ Sex ▪ Housing situation (living alone; with another person) ▪ Highest educational qualification Clinical characteristics (4 items) ▪ Years of diagnosis of AF (in years) ▪ Experience in pulse self-palpation (yes, no) ▪ Presence of an electronic device for pulse measurement at home (yes, no) ▪ Regular implementation of pulse self-measurement at home (no, yes [multiple times a day; once per day; not daily, but multiple times per week; not every week, but multiple times per month; not every month, but multiple times per year])	T_1_; IG and UCG	
Acceptability of intervention	Q2.A.I	*Self-reported questionnaire* Assessment of appropriateness of the intervention (7 items) ▪ Usefulness of intervention (5-point Likert-scale, very useful–not useful) ▪ Importance of learning PSP when suffering arrhythmias (5-point Likert-scale, very important–not important) ▪ Liking of intervention (5-point Likert-scale, very much–not at all) ▪ Would participants recommend the intervention to others (yes, no) ▪ Appraisal of duration of intervention (too short, accurate, too long) ▪ Appraisal of difficulty level of intervention (5-point Likert-scale, very easy–very difficult) ▪ Appraisal of comprehensibility of content of intervention (5-point Likert-scale, very easy to understand–very difficult to understand)	T_1_ (directly after intervention); IG	The PSPAF intervention was considered acceptable to intervention participants when: − ≥ 75% will rate the intervention partly to very reasonable − ≥ 75% will rate the intervention partly to very important − ≥ 75% will like the intervention partly to very much − ≥ 75% will eventually or definitively recommend the intervention to others − ≥ 75% will rate the duration of the intervention as accurate − ≥ 75% will rate the difficulty level of the intervention partly to very easy − ≥ 75% will rate the comprehensibility of the intervention as partly to very high − ≥ 75% will rate the intervention partly to very helpful − ≥ 90% did not experience any negative consequences related to the intervention − ≥ 75% will rate implementation of the content of the intervention in daily life partly to very easy − ≥ 75% will rate the likelihood of continuing a daily PSP twice a day as likely to very likely − ≥ 75% will be considered adherent to PSP (> 80% of possible entries are filled out)
	Q2.A.II	*Self-reported questionnaire* Assessment of perceived effectiveness (1 item) ▪ Extent of how helpful the intervention was concerning dealing with AF in everyday life (5-point Likert-scale, very helpful–not helpful) Assessment of perceived disadvantages (1 item) ▪ Negative consequences of intervention (yes, no, do not know) Assessment of perceived suitability (1 item) ▪ Difficulty level of implementation of the content of the intervention in everyday life (5-point Likert-scale, very easy–very difficult) Assessment of willingness of implementation (1 item) ▪ Likelihood of continuing a PSP twice a day (5-point Likert-scale, very likely–very unlikely)	T_2_; IG	
	D1	*Pulse diary* Assessment of adherence to PSP Booklet consisting of a table with 35 lines and 10 columns. Each line represents a day and has two fields for indications of time of day, heart rate and regularity or irregularity of the measured pulse, respectively. A total of 70 pulse measurements could be recorded. An additional field was implemented for any specifics or further measurements.	T_1_-T_2_; IG	
Feasibility of the intervention	F2	*Field note form* Context/resources Boxes assessing date, location, the existence of enough suitable rooms for delivering the intervention, and the presence of relatives during the delivery of the intervention	T_1_ (directly after intervention); IG	The PSPAF intervention was considered feasible when: − The intervention can be delivered within 30 min, − Fidelity to the intervention protocol will maintain at 85% − Appropriate and enough room will exist in ≥ 85% of sessions
	Audio recording	*Audio recordings of intervention sessions* Fidelity of intervention implementation (3 items) ▪ Rating of clarity, distinctness, and comprehensiveness of language, and verbalizations of interventionist (each item, 4-point rating scale; not at all (0 points)–completely (3 points); single weighting) ▪ Rating whether the whole content and all components have been delivered as listed in the intervention manual (4-point rating scale, not at all (0 points)–completely (3 points), threefold weighting) ▪ Rating whether the whole content and all components have been delivered in the correct order as listed in the intervention manual (4-point rating scale, not at all (0 points)–completely (3 points), threefold weighting) Time needed for delivering the intervention ▪ Assessed by the length of audio recordings (minutes, seconds)	T_1_; IG	
Feasibility of research methods	F1	*Field note form* (set up for every screening session) Process of recruitment (7 criteria) ▪ Date ▪ Location ▪ Amount of screened individuals ▪ Amount of eligible individuals ▪ Amount of excluded individuals ▪ Amount of included individuals Reasons for refusal ▪ Free text (indication was voluntary)	Stage of screening;Study coordinator	Research methods was considered feasible when: − Recruitment of the target number of participants (*n* = 20) was accomplished within 4 months of beginning the study − ≥ 80% of intervention participants attended the follow-up session (home visit) − Attrition will be ≤ 15% − ≥ 80% of data sets were completed − Treatment contamination occurred in ≤15% − ≥90% of participants perceived no or low burden of study participation
	C0	*Checklist* Process of recruitment and documentation of eligibility criteria Assessment of occurrence of each in- and exclusion criterion for every screened individual	Stage of screening; Study coordinator	
	F3	*Field note form* Resources and management (5 criteria) ▪ Date ▪ Number of homes visits (on that day) ▪ Amount of driven kilometers (on that day) ▪ Amount of time (minutes) required for home visits and travel routes (on that day) ▪ Specifics (free text)	T_2_; IG and UCG study assistant	
	Q2.C	*Self-reported questionnaire* Assessment of treatment contamination (3 items) ▪ Participation in a training to learn PSP 3–4 weeks since inclusion in study (yes, no, do not know) ▪ Reading or watching media reports containing a training to learn PSP 3–4 weeks since inclusion in study (yes, no, do not know) ▪ Advice or training to learn PSP through family physician 3–4 weeks since inclusion in study (yes, no, do not know)	T_2_; UCG	
Burden of study participation	Q2.B	*Self-reported questionnaire* Burden of study participation (1 item) ▪ Grading the statement “I felt burdened by participating in this study.” (5-point Likert-scale, totally agree–totally disagree, translated into very high–no burden) Subscale: if burden was perceived ➔ specification of kind of burden (3 items): psychological and/or physiological and/or financial burden	T_2_; IG and UCG	
Capability of PSP	C1	*Checklist* (for structured observation by study assistant) Structure (2 items) ▪ PSP in rest (correct, wrong) ▪ Use of a clock with second hand (correct, wrong) Process (5 items) ▪ Location of measurement (correct, wrong) ▪ Technique of measurement (correct, wrong) ▪ Duration of the measurement (correct, wrong) ▪ Stating determined value of the heart rate (no, yes [value in bpm]) ▪ Stating determined value of the heart rhythm (no, yes [rhythmic, arrhythmic]) Outcome (2 items) ▪ Comparison of heart rate determined by the participant and heart rate determined by mobile ECG device (bpm) [verification of the value after consultation with a physician] (correct, wrong—heart rate was considered correct if heart rate measured by participants was ± 8 bpm in comparison to the ECG) ▪ Comparison of heart rhythm determined by the participant and heart rhythm determined by mobile ECG device [ECG evaluation by a physician, rhythmic/arrhythmic] (correct, wrong—heart rhythm was considered correct if the measurement of participants was equal to the evaluation of a physician)	T_2_; IG and UCG	A participant is seen as capable of PSP if all 9 components are correct or fulfilled, respectively.
		*Mobile ECG device* ME 90; Beurer GmbH, Ulm	T_2_; IG and UCG	
Sign and symptom management	V1-3/Vs1-3	*Vignettes* (in written form) V1—physiologic values, V2—mild pathologic values, V3— severe pathologic values Sign and symptom management was rated “correct” or “wrong” by a study assistant by means of a standard solution for each vignette (Vs1-3).	T_2_; IG and UCG	Participants were considered being able to manage signs and symptoms if they solved the vignette correctly.

*AF* atrial fibrillation; *ECG* electrocardiogram; *IG* intervention group; *UCG* usual care group; C0, Q1, Q2, F1, F2, C1, V1-3, Vs1-3 = assessment instruments; *PSPAF* pulse self-palpation for patients with atrial fibrillation-intervention; *PSP* pulse self-palpation; *T1, T*_*2*_ = time points

#### Participant characteristics

Various socio-demographic and clinical characteristics were assessed via a self-reported questionnaire (Q1).

#### Intervention acceptability

Acceptability was operationalized into five attributes [61]: (1) appropriateness, (2) perceived effectiveness, (3) perceived disadvantages of the intervention, (4) suitability, (5) willingness, and (6) adherence. Attributes 1 to 5 were measured through a self-reported questionnaire consisting of two parts (Q2.A.I and II) containing 11 items in total and that were framed based on the suggestions of Francisco and Butterfoss [65]. Adherence to PSP was measured based on the number of entries in a pulse diary (D1) relative to the individual period between the date of intervention and the home visit. This was calculated as the number of entries presented divided by the number of possible entries. A participant was considered to be adherent to PSP if ≥ 80% of the possible entries in the pulse diary were filled out.

#### Intervention feasibility

Feasibility was operationalized into three components [61]: (1) context/resources (i.e., the existence of enough suitable rooms for delivering the intervention), (2) fidelity of intervention implementation (i.e., clarity, comprehensiveness, and logical sequencing of the information given to patients), and (3) time needed for delivering the intervention. Context/resources were determined through field notes (F2). In order to assess the fidelity of intervention implementation and time for interventions, every session was audiotaped. Recordings were then rated by the principle investigator.

#### Research method feasibility

Feasibility of research methods was determined with regard to three domains according to Thabane et al. [66]: (1) process, (2) resources, and (3) management. The domain process was comprised of the recruitment and data collection processes. It was assessed through field notes (F1) and a checklist (C0). Additionally, data were collected on attrition. The extent of treatment contamination [67] was assessed through a self-reported questionnaire (Q2.C). Resources and management domains were assessed by collecting data on the time spent for recruitment and on the field notes made by study assistants during home visits (F3).

#### Burden of study participation

Perceived burden of study participation was operationalized into the domains of psychological, physiological, and financial burden [68] and was assessed through a self-reported questionnaire (Q2.B).

#### Capability and sign-and-symptom management

Capability of performing PSP was operationalized into nine components, where all had to be fulfilled in order to rate a PSP as correctly performed. These components referred to the three dimensions of quality, i.e., structure, process, and outcome [69]. Capability was measured through a checklist (C1) and a mobile ECG device (ME 90; Beurer GmbH, Ulm).

Sign-and-symptom management was operationalized as the ability to correctly evaluate and respond to signs and/or symptoms of AF and was assessed using vignettes. Vignettes “comprise stimuli that selectively portray elements of reality to which research participants are invited to respond” (p. 918) and can appear in different forms [70]. They represent a practical, ethical, and cost-effective method to generate data [71] and can be used to assess perceptions, attitudes, and behaviors [70], and were used in the context of self-care decision-making [72]. For the purpose of this study, three different vignettes (V1–3) in written form were developed by the principle investigator and the study coordinator taking into account the recommendations of Hughes and Huby [73]. Each vignette briefly described a situation that contained a statement to heart rate and partly to symptoms in different severity levels (V1—physiologic values, V2—mild pathologic values, V3—severe pathologic values) and concluded with the question how participants would behave in this situation. Only one vignette at a time was presented to the participants for processing. The simultaneous processing of all three vignettes by all participants would have made it possible to compare them and thus facilitate their solution.

Vignettes were allocated during the randomization process under the proviso of an equal distribution in each group. In each group, V1 and V3 existed three times and V2 existed four times in a random sequence. Participants were considered capable of managing signs and symptoms if they correctly solved the vignette relative to a standard solution (Vs1–3).

### Procedures and data collection

Recruitment and follow-up took place between September 2017 and March 2018. After consent and randomization, baseline data were collected for all study participants (data collection point 1 = T_1_). Hereafter, participants in the IG received the PSPAF intervention and the pulse diary. After the session, participants in the IG filled out questionnaire Q2.A.I. Additional field notes (F2) were recorded by the study coordinator following each session. Participants in the UCG received care as usual, i.e., no education on pulse self-palpation.

**Fig. 1:**
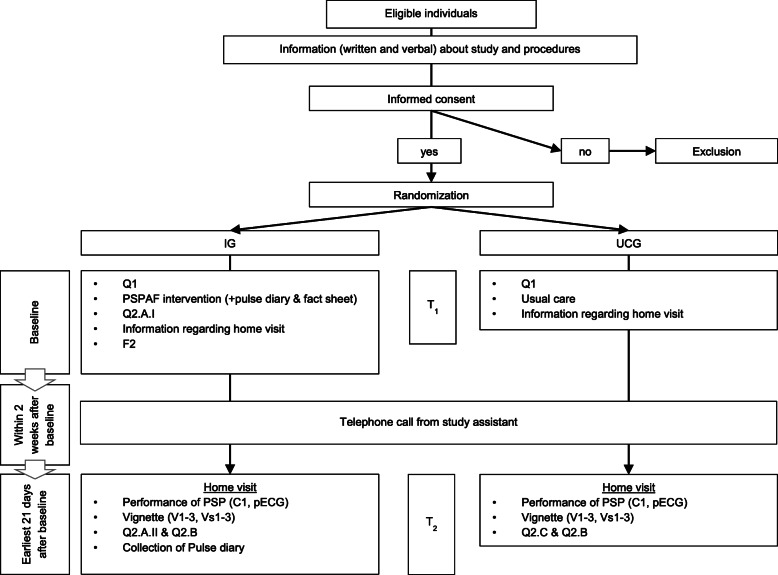
Flowchart of study procedures. IG intervention group, UCG usual care group; C0, Q1, Q2, F1, F2, C1, V1-3, Vs1-3. pECG = assessment instruments; T_1_, T_2_ = time points

In the first 2 weeks after enrollment, all participants received a phone call from a study assistant to schedule an appointment for a home visit (data collection point 2 = T_2_) within a period of 3–5 weeks after the intervention. Prior to the home visit, questionnaires addressing the acceptability of the intervention and the burden of study participation (IG) or on treatment contamination and the burden of study participation (UCG) were mailed to participants. A study assistant blinded to group allocation performed all home visits. During these visits, participants of both groups were asked to perform a PSP and their capability was assessed followed by an ECG recording using the mobile device. The assigned vignette was then presented to participants and they were asked to tell which action they would take in the depicted situation. The answer given was then rated by the study assistant with respect to the standard solution (Vs1–3). Finally, pulse diaries (only IG) and questionnaires were collected. After every home visit, the study assistant filled out a field note form (F3). A flowchart of the study procedures is provided in Fig. 1.

### Data analysis

Data were entered by the study coordinator. An independent study assistant randomly selected 50% of datasets and screened them for data entry accuracy prior to analysis. Potential entry errors were counted and corrected. A rate of entry errors greater than 10% would have had resulted in a complete revision and review of all data sets. The data were analyzed according to the intention-to-treat principle using IBM SPSS Statistics for Windows, version 22 (IBM Corp., Armonk, N.Y., USA) and Microsoft Excel 2013 (Microsoft, Redmond, Washington, USA).

Frequencies and percentages were calculated for all variables as appropriate. Means and standard deviations (SD) were calculated for normally distributed data. Medians (Mdn) and interquartile ranges (IQR) were calculated for non-normally distributed data. Validation of the acceptability and feasibility of the intervention, as well as of the feasibility of research methods was ascertained by predefined cut-off points (Table 2). Effect size estimates were calculated for capability and sign-and-symptom management and expressed as relative risks (RR) with 95% confidence intervals (CI) and as number needed to treat (NNT). In case of a value of zero in any cell of the contingency tables, we added plus one to every cell to allow for RR calculation. Missing values were descriptively summarized and their pattern was analyzed using Little’s Missing Completely at Random test. Cases with missing data were pairwise deleted in the respective statistical analysis.

## Results

### Sample

**Fig. 2:**
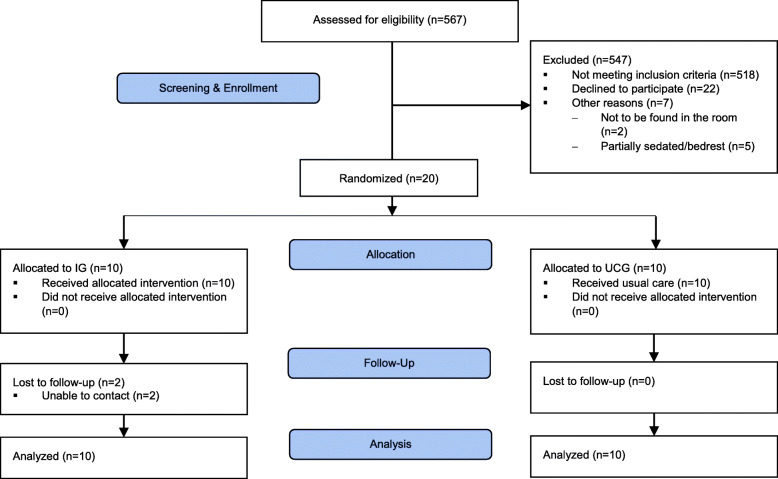
Participant flow diagram (referring to the CONSORT-Statement [53]). IG intervention group, UCG usual care group

For this study, a consecutive sampling approach was used. Figure 2 shows participant flow throughout the trial in a diagram according to the CONSORT statement extension to randomized pilot and feasibility trials [53]. Details on screening and recruitment are described later in the “Feasibility of research methods” section. The sample consisted of 20 individuals (70% male) with a mean age of 68.1 years (SD = 10.3; range = 43–84). Time since the diagnosis of AF ranged from 0.04 to 24 years (Mdn = 2.6; IQR = 0.2–8.8). Thirteen participants (65%) were living with another person in the same household. Sixteen participants (80%) held either a primary or a secondary school degree. Eight participants (40%) stated having experiences with PSP and 16 (80%) possessed an electronic device for pulse measurement. Six participants (30%) measured their pulse once or multiple times a day, whereas a quarter of participants (*n* = 5) had never measured their pulse at home (Table 3).

**Table 3:** Demographic and clinical characteristics of study groups

	IG (*n* = 10)	UCG (*n* = 10)
Mean age in years (SD, range)	65.8 (11.2, 43.0–78.0)	70.3 (9.3, 55.0–84.0)
Median number of years with diagnosis of AF (IQR, range)	0.9 (0.1–8.5, 0.04–20.0)	4.0 (0.8–10.0, 0.06–24.0)
Sex	*n* (%)	
Female	2 (20%)	4 (40%)
Male	8 (80%)	6 (60%)
Housing situation		
Living alone	5 (50%)	2 (20%)
Living together with another person	5 (50%)	8 (80%)
Highest educational qualification		
None	0 (0%)	0 (0%)
Primary/main school	4 (40%)	4 (40%)
Secondary school	4 (40%)	4 (40%)
Polytechnic school	0 (0%)	0 (0%)
High school	1 (10%)	2 (20%)
University	1 (10%)	0 (0%)
Existing experience in PSP		
Yes	3 (30%)	5 (50%)
No	7 (70%)	5 (50%)
Presence of an electronic device for pulse measurement at home		
Yes	7 (70%)	9 (90%)
No	3 (30%)	1 (10%)
Regular implementation of pulse self-measurement at home		
No	3 (30%)	2 (20%)
Multiple times a day	0 (0%)	1 (10%)
Once per day	3 (30%)	2 (20%)
Not daily, but multiple times per week	3 (30%)	1 (10%)
Not every week, but multiple times per month	1 (10%)	2 (20%)
Not every month, but multiple times per year	0 (0%)	2 (20%)

*IG* intervention group, *IQR* interquartile range, *PSP* pulse self-palpation, *SD* standard deviation, *UCG* usual care group

### Acceptability of the intervention

Immediately after the intervention (T_1_), participants of the IG (*n* = 10) rated it as follows: very useful (50%), rather useful (40%), or partly useful (10%) and also very important (80%) or rather important (20%). The intervention was rather liked (30%) or very much liked (60%) and all participants of the IG stated they would recommend it to others. The intervention was rated as having an accurate duration (100%), an easy level of difficulty (90%), and as easy to comprehend (100%).

At T_2_, five participants (71.4%) of the IG (*n* = 7) perceived the intervention as being helpful or very helpful in dealing with AF in everyday life. No participant perceived negative consequences in relation to the intervention. However, one participant (14.3%) did not know whether a negative consequence was perceived or not. For 57.1% of the IG, the intervention was very easy to implement into daily life. One participant (14.3%) perceived it as partly easy/difficult, whereas two participants (28.6%) had great difficulties with the implementation into daily life. The self-reported probability of continuing a daily PSP twice a day was likely or very likely for 71.4% of the IG. All negative ratings on T_2_ were made by the same individuals.

**Fig. 3:**
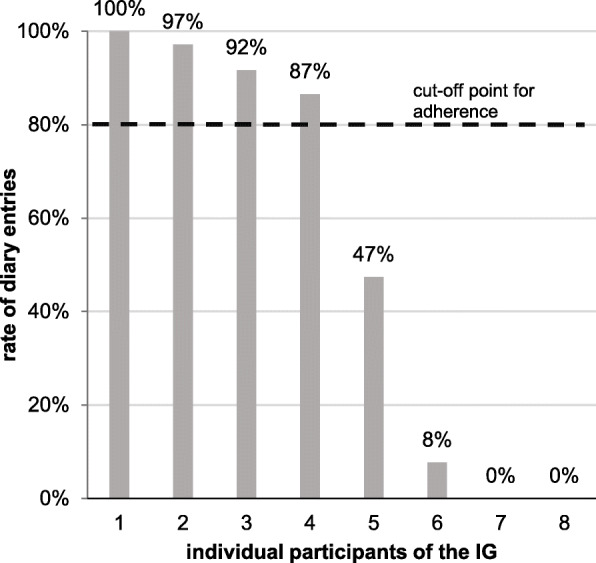
Adherence to PSP in the IG. IG intervention group, PSP pulse self-palpation

Eight pulse diaries (80%) of the participants could be collected. The median time between intervention and home visit to observe the adherence to PSP was 26 days (IQR = 25.5–32.0; range = 19–54). Four participants (50%) filled out 87–100% of possible diary entries and were therefore considered to be adherent to PSP. Four participants (50%) were considered to be non-adherent because only 0–47% of possible diary entries were completed (Fig. 3). Seven out of the 12 preliminary cut-off points to validate acceptability of the intervention were reached (Table 4).

**Table 4:** Acceptability of the PSPAF intervention and cut-off points

Preliminary cut-off points	Threshold (%)	Observed values (%)	Cut-off point reached?
The PSPAF intervention was considered acceptable when participants…			
1. Rated the intervention partly to very useful	≥ 75	100	Yes
2. Rated the intervention partly to very important	≥ 75	100	Yes
3. Liked the intervention partly to very much	≥ 75	100	Yes
4. Will eventually or definitively recommend the intervention to others	≥ 75	100	Yes
5. Rated the duration of the intervention as accurate	≥ 75	100	Yes
6. Rated the difficulty level of the intervention partly to very easy	≥ 75	100	Yes
7. Rated the comprehensibility of the intervention as partly to very high	≥ 75	100	Yes
8. Rated the intervention partly to very helpful concerning dealing with AF in everyday life	≥ 75	71.4	No
9. Did not experience any negative consequences related to the intervention	≥ 90	85.7	No
10. Rated implementation of the content of the intervention in daily life partly to very easy	≥ 75	71.4	No
11. Rated the likelihood of continuing a daily PSP twice a day as rather likely to very likely	≥ 75	71.4	No
12. Were considered adherent to PSP (> 80% of possible entries were filled out)	≥ 75	50.0	No

*PSP* pulse self-palpation, *PSPAF* pulse self-palpation for patients with atrial fibrillation intervention

### Feasibility of the intervention

No relative was present during any of the intervention sessions. The median duration of the interventions was 17:41 min (IQR = 15:40–19:19; range = 11:07–24:42). The median rate of the fidelity of intervention implementation was 92.6% (IQR = 85.2–96.3; range = 70.4–100). In eight of the intervention sessions, minor comprehension limitations were observed due to the use of technical terms by the interventionist. In two sessions, the cut-off rate (85%) was not reached due to comprehension problems in combination with minor deviations in content. All preliminary cut-off points to validate the feasibility of the intervention were reached (Table 5).

**Table 5:** Feasibility of the PSPAF intervention and cut-off points

Preliminary cut-off points to validate the feasibility of the intervention	Threshold	Observed values	Cut-off point reached?
1. Maximum timeframe for delivering the intervention	30 min	17:41^a,b^ (max = 24:42^b^)	Yes
2. Rate of fidelity to the intervention protocol	≥ 85%	92.6%^a^	Yes
3. Rate of intervention sessions with appropriate and enough room	≥ 85%	100.0%	Yes

*max* maximum, *PSPAF* pulse self-palpation for patients with atrial fibrillation-intervention

^a^Median

^b^Minutes:seconds

### Feasibility of research methods

#### Recruitment process

Recruitment of participants was accomplished on 16 dates, with a respective minimum interval of 7 days between dates. In order to reach the target number of participants, the recruitment phase lasted 134 days, exactly 12 days longer than the cut-off period of 4 months.

A total of 567 electronic health records or individuals were screened and assessed for eligibility. Of these, 525 individuals (93%) were ineligible to participate in the trial. Of 42 eligible patients, 20 consented to become participants, representing a recruitment rate of 50% (Fig. 1). The main reasons for refusal were (1) concerns that participation would be too burdensome, (2) no interest or doubts about the meaningfulness of the intervention, or (3) organizational reasons (i.e., immediate discharge or transfer).

#### Eligibility criteria

The three most common inapplicable inclusion criteria were “living within a 30 min car ride to the participating health care center” (449 times, 79.2%), “diagnosis of paroxysmal or persistent AF” (279 times, 49.2%), and “being able to read, write and understand German” (66 times, 11.6%). Moreover, 174 individuals (30.7%) were excluded due to living outside the predefined geographical area. The most commonly occurring exclusion criteria were “physical impairment of both hands” (22 times, 3.9%) and “cognitive impairment” (19 times, 3.4%). Additionally, 19 patients (3.4%) were excluded because of reasons not described in the exclusion criteria. These reasons could be summarized in two categories: (1) patient is sedated or on bedrest and/or (2) patient could not be found in his/her room.

#### Follow-up and missing data

Overall, 18 participants (90%) attended the home visit at T_2_. Two participants of the IG were unavailable for scheduling a home visit, representing an attrition rate of 10%. Another participant from the IG did not complete the questionnaires on T_2,_ but took part in the home visit. During a visit to a UCG participant, no ECG could be recorded due to technical difficulties. Complete data sets were available for 16 participants (80%) (IG, 7/70%; UCG, 9/90%). Ultimately, there were no missing data for T_1_, and at T_2_, the rate of missing data ranged from 10 to 30%. There was no consistent pattern for missing data.

#### Treatment contamination

Treatment contamination could not be observed. During the study period, no participant of the UCG (*n* = 10) took part in a PSP training, read or watched any media containing elements of PSP training, or received advice and/or training from a general practitioner to learn PSP.

#### Resources and management

During recruitment, the study coordinator had a time exposure of 3–5 h per date of recruitment. Study assistants accomplished 18 home visits. In total, study assistants covered 416 km (Mdn = 35; IQR = 16.3–70.5; range = 1–83) and had an overall time exposure of 1205 min (Mdn = 110; IQR = 90–148; range = 40–180) for the home visits.

### Burden of study participation

Overall, 15 out of 17 participants (88.2%) experienced low or no burden throughout study participation. In the UCG (*n* = 10), no (70%) or a low burden was perceived. In the IG (*n* = 7), one participant experienced a very high burden (psychological and physiological) and another participant reported a partly (psychological) burden. No participant reported a perceived financial burden. Four out of the six preliminary cut-off points to validate the feasibility of research methods were reached at T_2_ (Table 6).

**Table 6:** Feasibility of research methods and cut-off points

Preliminary cut-off points to validate the feasibility of research methods	Threshold	Observed values	Cut-off point reached?
Recruitment of the target number of participants (*n* = 20) within 4 months of study initiation (= 122 days)	122 days	134 days	No
Attendance of intervention participants in follow-up sessions (home visits)	≥ 80%	90.0%	Yes
Attrition rate	≤ 15%	10.0%	Yes
Rate of complete data sets	≥ 80%	85.0%^a^	Yes
Rate of treatment contamination	≤ 15%	0.0%	Yes
Rate of participants having perceived no or low burden of study participation	≥ 90%	88.2%	No

*IG* intervention group, *UCG* usual care group

^a^IG, 8/80% and UCG, 9/90%

### Capability

IG and UCG differed in terms of the capability of PSP. Four participants (50%) of the IG (*n* = 8) and no participant of the UCG (*n* = 10) were considered capable of performing PSP. This resulted in a RR of 6.0 (95% CI = [0.8, 43.3]) and a NNT of 2.4. When comparing individual components of a PSP, the highest RR values were observed for the reporting of the determined value of the heart rhythm, and in determining heart rate and rhythm. The lowest RR values were observed for performing a PSP in the rest state and in reporting the determined value of the heart rate (Table 7).

**Table 7:** Group comparison regarding capability of PSP

Components of PSP	Rating	IG (*n* = 8) *n* (%)	UCG (*n* = 10) *n* (%)	RR (95% CI)
1) PSP in rest	Correct	5 (62.5)	4 (40.0)	1.6 (0.6–3.9)
	Wrong	3 (37.5)	6 (60.0)	
2) Use of a clock with second hand	Correct	6 (75.0)	3 (30.0)	2.5 (0.9–6.9)
	Wrong	2 (25.0)	7 (70.0)	
3) Location of measurement	Correct	6 (75.0)	4 (40.0)	1.8 (0.8–4.4)
	Wrong	2 (25.0)	6 (60.0)	
4) Technique of measurement	Correct	6 (75.0)	2 (20.0)	3.8 (1.0–13.8)
	Wrong	2 (25.0)	8 (80.0)	
5) Duration of the measurement	Correct	6 (75.0)	2 (20.0)	3.8 (1.0–13.8)
	Wrong	2 (25.0)	8 (80.0)	
6) Reporting determined value of the heart rate	Yes	5 (62.5)	4 (40.0)	1.6 (0.6–3.9)
	No	3 (37.5)	6 (60.0)	
7) Reporting determined value of the heart rhythm	Yes	4 (50.0)	0 (0.0)	6.0 (0.8–43.3)
	No	4 (50.0)	10 (100.0)	
8) Determined heart rate^a^	Correct	5 (62.5)	1 (11.1)	5.6 (0.8–38.5)
	Wrong	3 (37.5)	8 (88.9)	
9) Determined heart rhythm^a^	Correct	4 (50.0)	0 (0.0)	5.5 (0.8–39.4)
	Wrong	4 (50.0)	9 (100.0)	
Overall PSP performance	Correct	4 (50.0)	0 (0.0)	6.0 (0.8–43.3)
	Wrong	4 (50.0)	10 (100.0)	

*IG* intervention group, *PSP* pulse self-palpation, *RR* relative risk, *UCG* usual care group

^a^Only nine cases were analyzed in UCG because of missing values due to technical problems with the mobile ECG

### Sign-and-symptom management

Six participants (75%) of the IG (*n* = 8) and five of the UCG (*n* = 10) solved the vignettes correctly, resulting in a RR of 1.5 (95% CI = [0.7, 3.1]) and a NNT of 4.0. Vignette V1 was solved correctly by all four participants (IG: *n* = 1; UCG: *n* = 3) and V3 was solved correctly by 83.3% (IG: *n* = 3; UCG: *n* = 2) of the participants (*n* = 6). Vignette V2 was answered incorrectly by 75.0% (IG: *n* = 2; UCG: *n* = 4) of the participants (*n* = 8).

## Discussion

In this pilot trial, the PSPAF intervention was tested with respect to acceptability and feasibility. Moreover, the feasibility of research methods and the burden of study participation were assessed in combination with the calculation of effect size estimates of PSP capability and sign-and-symptom management. In summary, the PSPAF intervention seemed to be feasible, but only partly acceptable. The feasibility of applied research methods highlighted some limitations. While estimations of effect sizes seemed to correlate to an effect of the intervention on the capability of performing PSP, the effect on sign-and-symptom management remains unclear.

### Acceptability and feasibility of the PSPAF intervention

The PSPAF intervention did not reach preliminary cut-off points at T_2_; therefore, it could only be considered to be partially acceptable. The intervention seemed appropriate and comprehensible and had no negative consequences for participants. Yet it showed limitations in its potential effectiveness, suitability, and in addressing participants’ willingness of implementing its content into daily life. The latter of which was also reflected in the rate of adherence to PSP. Two individuals experienced difficulties with including the intervention into daily life, resulting in zero pulse diary entries. The lack of one-third of the data at T_2_ on this outcome further hinders a final judgment.

Overall, adherence to PSP was heterogenous, and two different groups could be observed. One group was adherent and the other was non-adherent to PSP. This differs from another pilot study [52] where 94% of participants filled out more than 88% of possible diary entries. Reasons for the lower rate in this study remain unclear as corresponding data were not collected. We hypothesize that self-efficacy (as a moderator and mediator of all elements of self-management [25]) and motivation (as an essential element of healthy behavior [74]) differed in participants, ultimately affecting their adherence. In addition, AF is associated with depression [8], which in turn could have negatively affected adherence to self-monitoring [75]. Another possibility is a lower perceived level of disease controllability, thus affecting the willingness for regular self-monitoring [76]. Ideally, the intervention should encompass these aspects.

The PSPAF intervention could be considered feasible. It was completed within a short period of time, underlying its practicability in health care settings. However, this is based solely on one pilot study in one local setting. The fidelity of intervention implementation was high. Nevertheless, minor limitations were observed despite the interventionist being a co-developer of the intervention with prior advanced knowledge of its mechanisms and content.

### Feasibility of research methods

Applied research methods could not be considered feasible because of a longer-lasting recruitment phase and an increased rate of burden throughout study participation.

The transgression of the originally defined recruitment phase was lower than 10% and might therefore be considered to be reasonable. The prolongation of the recruitment phase was due to delays in recruitment: numerous holidays during the recruitment phase and a large number of ineligible patients further highlight recruitment as one of the most common challenges in randomized controlled trials [77]. Interestingly, other studies with similar interventions showed lower recruitment rates (15–33%) [44, 51, 52]. In our trial, one-third of eligible individuals had to be excluded due to living outside the predefined geographical area suggesting the criterion to be too restrictive. However, given the constraints of this pilot study, including limited financial and personnel resources, this could not have been altered. Additionally, only half of eligible participants consented to study participation. The assessment of reasons for refusing participation aided in understanding why potential participants declined [78]. These reasons taken together, suggest that the potential disadvantages of participation outweighed the benefits when deciding to participate [79]. Furthermore, besides the supposition of a potential burden, some eligible individuals were concerned about the meaningfulness of the intervention. Similar to the assumption of Koller et al. [80], the latter could be partially attributed to the fact that clinical nursing research in Germany, especially at the study site, is still quite novel. Patients are not familiar with this branch of science and could have been reluctant to participate. Adaptations and improvements of recruitment strategies could perhaps change presuppositions of potential participants towards perceiving studies to be more beneficial, which could enhance recruitment rates [77, 79, 81].

Two participants of the IG experienced a notable psychological and physiological burden throughout study participation. A comparable pilot study found a low rate of participant burden [52]. However, the burden was not specified as it was in our study and the participants obtained monetary incentives for several data collection points, potentially altering their experience. Due to the subjective nature of perceiving burden [68] and since we did not further investigate detailed reasons for these kinds of burden, we can only speculate about potential reasons.

In addition, the possible influence of the intervention must be considered. Although no participant experienced negative consequences from the intervention, there could have been an effect on their perceptions. In the literature, the following possible reasons are mentioned: proactive involvement with AF in terms of self-management was distressing [82], and too much time and cognitive efforts were required [64]. It must also be considered that the two participants of the IG who were lost to follow up did not continue participation because of burden. Although definitive reasons for the burden of study participation remain unknown, there are indications for potential burden that need to be monitored in a future trial.

### Effect size estimates

The probability of being capable of PSP was six times greater in the IG compared to the UCG. The RR suggests a large effect of the PSPAF intervention [83] concerning the capability of carrying out PSP that is considered to be clinically significant [84]. However, the broad confidence interval reveals the inaccuracy of this value and by including the value 1, there is a risk of no effect at all. This also applies to the individual components of a PSP. The small NNT [85] indicates that on average, two patients would have had to receive the PSPAF intervention (instead of usual care) for at least one additional patient to be capable of performing PSP. We might therefore assume, albeit with caution, that the intervention could have improved this capability, which is consistent with the findings of other studies with similar interventions [38, 44, 45].

With respect to sign-and symptom-management, group differences were smaller. The probability of correctly solving the vignette was 50% greater in the IG compared to the UCG. The RR suggests almost no effect of the PSPAF intervention concerning sign-and-symptom management knowledge [83], but it could also be considered to be clinically significant [84]. Furthermore, the confidence interval includes the value 1, which in turn implies no effect. The NNT indicates that on average, four patients would have had to receive the PSPAF intervention (instead of usual care) for at least one additional patient to show correct knowledge about sign-and-symptom management. Although this ratio seems to be acceptable [85], the effect of the intervention seems questionable. Other studies have investigated a similar outcome and observed greater effects but our findings are hardly comparable due to differences in operationalization [52] and/or methodology [38].

## Limitations

The present study has several limitations. Consecutive sampling and restrictive eligibility criteria reduced the external validity of our results [86]. Furthermore, the small sample size, which might be reasonable for a pilot study [87], undermines statistical conclusion validity [88]. Missing data and the practice of pairwise deletion further contribute to this fact. In our study, it mostly affected the determination and accuracy of effect sizes, which in any case tend to be larger in pilot trials than in a definitive trial [88]. Although pilot trials do not provide meaningful effect sizes [89], it is possible to investigate the potential mechanisms of efficacy for a new intervention [87]. Therefore, only an estimation of effect sizes has been intended in our study but the results must be regarded as preliminary and interpreted with caution.

Another limitation is the exclusive use of newly developed instruments for data collection without having information regarding the psychometric properties based on systematic analysis. Although the instruments may be considered to be face valid [90], and verbal probing as well as think-aloud interviews can strengthen content validity and the reliability of instruments [64], they cannot be considered to be fully valid or reliable. This results in the potential of imprecise measurements, which ultimately have implications on statistical conclusion validity [91]. Above all, drawing inferences from diary entries about adherence remains uncertain as evidence indicates that some patients do not use diaries despite recommendations [92]. The same is true for the use of vignettes as indicators of sign-and-symptom management. Despite their advantages in examining judgments and decisions [93], their accuracy is unknown and questionable [88] and, thus, may have biased the results.

Lastly, it cannot be confirmed that the blinding of study assistants was always maintained. Study participants may have imparted their group appointment to the assistant and/or assistants may have drawn conclusions due to improperly packed documents collected at T_2_. These circumstances may have led to reduced objectivity of study assistants’ judgements [94], and possibly resulted in incorrect ratings.

## Implications

Based on the results of this study, we formulate the following suggestions and recommendations regarding the PSPAF intervention with a possible progression to a future definitive trial.

The intervention could be enhanced with encouraging elements to improve the motivation and adherence of participants. Using elements of motivational interviewing [74, 95], stressing the advantages of the intervention and the diary [92] and encouraging an increased involvement of relatives [75] could be conceivable. Furthermore, the use of telephone calls shortly after the intervention should be considered as a means to identify and address difficulties of participants and facilitating implementation and adherence [75].

In order to allow for a larger sample, the inclusion criterion limiting the geographical area should be adjusted. Recruitment should be performed at different centers, and recruitment strategies optimized through adaptation of the written study information and personnel training. At last, the relocation of the follow-up assessment to a central location in combination with incentives for participants should be considered in order to decrease the number of or complete avoidance of home visits.

Participant burden should be monitored. The aforementioned telephone call would offer the possibility to identify and reduce patient burden. This topic will be best addressed in a future qualitative sub-study to explore the specific burden of study participants.

The instruments assessing the main study outcomes (capability, sign-and-symptom management, adherence) must be evaluated for their validity and reliability, so as to ensure precise measurements and valid statistical calculations. The fidelity of intervention implementation has to be ensured through careful training of interventionists [96] and should optimally be monitored continuously by two experts (rater, interrater), in order to warrant a high level of objective ratings and a good quality of intervention implementation.

Procedures to support study blinding have to be strictly observed in order to reduce the risk of bias. Data collectors require careful briefing on this topic and study participants need to be urged to not communicate information regarding group appointment and/or received intervention.

Further investigations should also include an analysis of correlations between demographic and clinical characteristics and outcomes, which could identify possible determinants for outcome measures, as observed in other studies [42, 44].

## Conclusions

Results of the present pilot trial contribute to a better understanding of the PSPAF intervention, afford a first impression of its possible effects, and provide information for planning a follow-up study. At large, the intervention appears to be promising and applicable and could be optimized with a few adjustments. Due to methodological restraints, the true effects of the intervention remain vague and need to be further examined in a fully powered trial. We recommend conducting a full-scale trial with respect to the suggestions and amendments mentioned above in an effort to ensure the benefit and efficacy of the PSPAF intervention. For planning and conducting such a trial, investigators can use the results obtained in this pilot trial.

## Supplementary information

**Additional file 1.** CONSORT extension for Pilot and Feasibility Trials Checklist for the PSPAF Pilot Trial.

## Data Availability

The datasets used and/or analyzed during the current study are available from the corresponding author on reasonable request.

## Abbreviations

AF: Atrial fibrillation
BCT: Behavioral change techniques
C0: Checklist to assess the feasibility of research methods
C1: Checklist to assess the capability of PSP
CI: Confidence interval
D1: Pulse diary (assessment of adherence to PSP)
ECG: Electrocardiogram
F1: Field note form to assess the feasibility of research methods
F2: Field note form to assess the feasibility of the intervention
F3: Field note form to assess the feasibility of research methods
IG: Intervention group
IQR: Interquartile range
max: Maximum
Mdn: Median
NNT: Number needed to treat
PSP: Pulse self-palpation
PSPAF intervention: Pulse self-palpation for patients with atrial fibrillation intervention
Q1: Self-reported questionnaire to assess demographic and clinical characteristics of participants
Q2.A.I: Self-reported questionnaire to assess the acceptability of intervention at T_1_
Q2.A.II: Self-reported questionnaire to assess the acceptability of intervention at T_2_
Q2.B: Self-reported questionnaire to assess the burden of study participation
Q2.C: Self-reported questionnaire to assess the feasibility of research methods
RR: Relative risk
SD: Standard deviation
T_1_: Data collection point 1
T_2_: Data collection point 2
UCG: Usual care group
V1-3: Vignettes to assess sign and symptom management
Vs1-3: Standard solutions for vignettes V1-3

## References

[1] Camm AJ, Kirchhof P, Lip GYH, Schotten U, Savelieva I, Ernst S (2010). Guidelines for the management of atrial fibrillation: the Task Force for the Management of Atrial Fibrillation of the European Society of Cardiology (ESC). Eur Heart J..

[2] Camm AJ, Lip GYH, Caterina RD, Savelieva I, Atar D, Hohnloser SH (2012). 2012 focused update of the ESC guidelines for the management of atrial fibrillation. Eur Heart J..

[3] Chugh SS, Havmoeller R, Narayanan K, Singh D, Rienstra M, Benjamin EJ (2014). Worldwide epidemiology of atrial fibrillation: a Global Burden of Disease 2010 Study. Circulation..

[4] Stewart S, Hart CL, Hole DJ, McMurray JJV (2002). A population-based study of the long-term risks associated with atrial fibrillation: 20-year follow-up of the Renfrew/Paisley study. Am J Med..

[5] Kirchhof P, Benussi S, Kotecha D, Ahlsson A, Atar D, Casadei B (2016). ESC Guidelines for the management of atrial fibrillation developed in collaboration with EACTS. Eur Heart J..

[6] Shah AD, Merchant FM, Delurgio DB (2016). Atrial fibrillation and risk of dementia/cognitive decline. J Atr Fibrillation..

[7] Thrall G, Lane D, Carroll D, Lip GYH (2006). Quality of life in patients with atrial fibrillation: a systematic review. Am J Med.

[8] Patel D, Mc Conkey ND, Sohaney R, Mc Neil A, Jedrzejczyk A, Armaganijan L. A systematic review of depression and anxiety in patients with atrial fibrillation: the mind-heart link. Cardiovasc Psychiatry Neurol. 2013;2013. 10.1155/2013/159850 PMC3655604 23710335

[9] Ball J, Carrington MJ, McMurray JJV, Stewart S (2013). Atrial fibrillation: profile and burden of an evolving epidemic in the 21st century. Int J Cardiol..

[10] Rohrbacker NJ, Kleinman NL, White SA, March JL, Reynolds MR (2010). The burden of atrial fibrillation and other cardiac arrhythmias in an employed population: associated costs, absences, and objective productivity loss. J Occup Environ Med Am Coll Occup Environ Med..

[11] Amin AN, Jhaveri M, Lin J (2013). Hospital readmissions in US atrial fibrillation patients: occurrence and costs. Am J Ther..

[12] Lundqvist CB, Lip GYH, Kirchhof P. What are the costs of atrial fibrillation? Europace. 2011:13:ii9–12. 10.1093/europace/eur087 21518753

[13] Steinberg BA, Kim S, Fonarow GC, Thomas L, Ansell J, Kowey PR (2014). Drivers of hospitalization for patients with atrial fibrillation: results from the Outcomes Registry for Better Informed Treatment of Atrial Fibrillation (ORBIT-AF). Am Heart J.

[14] Singh SN (2012). Costs and clinical consequences of suboptimal atrial fibrillation management. Clin Outcomes Res CEOR..

[15] Aliot E, Breithardt G, Brugada J, Camm J, Lip GYH, Vardas PE (2010). An international survey of physician and patient understanding, perception, and attitudes to atrial fibrillation and its contribution to cardiovascular disease morbidity and mortality. Europace..

[16] Vizzardi E, Curnis A, Latini MG, Salghetti F, Rocco E, Lupi L (2014). Risk factors for atrial fibrillation recurrence: a literature review. J Cardiovasc Med Hagerstown Md..

[17] Calkins H, Reynolds MR, Spector P, Sondhi M, Xu Y, Martin A (2009). Treatment of atrial fibrillation with antiarrhythmic drugs or radiofrequency ablation. Circ Arrhythm Electrophysiol..

[18] Qin D, Leef G, Alam MB, Rattan R, Munir MB, Patel D (2016). Comparative effectiveness of antiarrhythmic drugs for rhythm control of atrial fibrillation. J Cardiol..

[19] Li H, Riedel R, Oldemeyer JB, Rovang K, Hee T (2004). Comparison of recurrence rates after direct-current cardioversion for new-onset atrial fibrillation in patients receiving versus those not receiving rhythm-control drug therapy. Am J Cardiol..

[20] Siu C-W, Jim M-H, Zhang X, Chan Y-H, Pong V, Kwok J (2009). Comparison of atrial fibrillation recurrence rates after successful electrical cardioversion in patients with hyperthyroidism-induced versus non-hyperthyroidism-induced persistent atrial fibrillation. Am J Cardiol..

[21] Ganesan AN, Shipp NJ, Brooks AG, Kuklik P, Lau DH, Lim HS (2013). Long-term outcomes of catheter ablation of atrial fibrillation: a systematic review and meta-analysis. J Am Heart Assoc..

[22] Wynn GJ, El-Kadri M, Haq I, Das M, Modi S, Snowdon R (2016). Long-term outcomes after ablation of persistent atrial fibrillation: an observational study over 6 years. Open Heart..

[23] McCabe PJ, Schad S, Hampton A, Holland DE (2008). Knowledge and self-management behaviors of patients with recently detected atrial fibrillation. Heart Lung J Crit Care..

[24] Wilkinson A, Whitehead L (2009). Evolution of the concept of self-care and implications for nurses: a literature review. Int J Nurs Stud..

[25] Richard AA, Shea K (2011). Delineation of self-care and associated concepts. J Nurs Scholarsh Off Publ Sigma Theta Tau Int Honor Soc Nurs..

[26] Grady PA, Gough LL (2014). Self-management: a comprehensive approach to management of chronic conditions. Am J Public Health..

[27] McGowan PT (2012). Self-management education and support in chronic disease management. Prim Care..

[28] Riegel B, Jaarsma T, Strömberg A (2012). A middle-range theory of self-care of chronic illness. Adv Nurs Sci..

[29] Hibbard JH, Greene J (2013). What the evidence shows about patient activation: better health outcomes and care experiences; fewer data on costs. Health Aff Proj Hope..

[30] Wilde MH, Garvin S (2007). A concept analysis of self-monitoring. J Adv Nurs..

[31] Dodd M, Janson S, Facione N, Faucett J, Froelicher ES, Humphreys J (2001). Advancing the science of symptom management. J Adv Nurs..

[32] Stedman TL (2012). Stedman’s Medical Dictionary for the Health Professions and Nursing.

[33] Sidani S, Doran DM (2011). Symptom management. Nurs Outcomes State Sci.

[34] Ekblad H, Rönning H, Fridlund B, Malm D (2013). Patients’ well-being: experience and actions in their preventing and handling of atrial fibrillation. Eur J Cardiovasc Nurs J Work Group Cardiovasc Nurs Eur Soc Cardiol..

[35] Hoppe UC (2011). Detektion von Vorhofflimmern beim Schlaganfall. Nervenarzt..

[36] Shea JB, Sears SF (2008). A patient’s guide to living with atrial fibrillation. Circulation..

[37] Ajijola OA, Boyle NG, Shivkumar K. Detecting and monitoring arrhythmia recurrence following catheter ablation of atrial fibrillation. Front Physiol. 2015;6. 10.3389/fphys.2015.00090 PMC4376077 25870562

[38] Munschauer FE, Sohocki D, Smith Carrow S, Priore RL (2004). A community education program on atrial fibrillation: implications of pulse self-examination on awareness and behavior. J Stroke Cerebrovasc Dis..

[39] National Clinical Guideline Centre (UK) (2014). Atrial fibrillation: the management of atrial fibrillation.

[40] Jones C, Pollit V, Fitzmaurice D, Cowan C (2014). The management of atrial fibrillation: summary of updated NICE guidance. The BMJ..

[41] Apostolidis P, Schmalstieg P. Puls. In: Lauber A, Schmalstieg P, editors. Verstehen und Pflegen 2: Wahrnehmen und Beobachten. 3rd ed. Stuttgart: Thieme; 2012. p. 130–40.

[42] Kallmünzer B, Bobinger T, Kahl N, Kopp M, Kurka N, Hilz M-J (2014). Peripheral pulse measurement after ischemic stroke - a feasibility study. Neurology..

[43] Harris K, Edwards D, Mant J (2012). How can we best detect atrial fibrillation?. J R Coll Physicians Edinb..

[44] Virtanen R, Kryssi V, Vasankari T, Salminen M, Kivelä S-L, Airaksinen KJ (2014). Self-detection of atrial fibrillation in an aged population: the LietoAF Study. Eur J Prev Cardiol..

[45] Munschauer FE, Hens MM, Priore RL, Stolarski E, Buffamonte S, Carlin A (1999). Screening for atrial fibrillation in the community: a multicenter validation trial. J Stroke Cerebrovasc Dis Off J Natl Stroke Assoc..

[46] Jaakkola J, Vasankari T, Virtanen R, Juhani Airaksinen KE (2017). Reliability of pulse palpation in the detection of atrial fibrillation in an elderly population. Scand J Prim Health Care..

[47] Desteghe L, Engelhard L, Raymaekers Z, Kluts K, Vijgen J, Dilling-Boer D (2016). Knowledge gaps in patients with atrial fibrillation revealed by a new validated knowledge questionnaire. Int J Cardiol..

[48] Dobreanu D, Svendsen JH, Lewalter T, Hernández-Madrid A, Lip GYH, Blomström-Lundqvist C (2013). Current practice for diagnosis and management of silent atrial fibrillation: results of the European Heart Rhythm Association survey. Europace..

[49] McCabe PJ (2011). What patients want and need to know about atrial fibrillation. J Multidiscip Healthc..

[50] Lubitz SA, Yin X, Rienstra M, Schnabel RB, Walkey AJ, Magnani JW (2015). Long-term outcomes of secondary atrial fibrillation in the community: the Framingham Heart Study. Circulation..

[51] Benito L, Coll-Vinent B, Gómez E, Martí D, Mitjavila J, Torres F, et al. EARLY: a pilot study on early diagnosis of atrial fibrillation in a primary healthcare centre. Europace. 2015;euv146. 10.1093/europace/euv146 26071233

[52] McCabe PJ, Douglas KV, Barton DL, Austin C, Delgado A, DeVon HA. Feasibility testing of the alert for AFib intervention. West J Nurs Res. 2016;0193945916656609. 10.1177/0193945916656609 PMC5347363 27387372

[53] Eldridge SM, Chan CL, Campbell MJ, Bond CM, Hopewell S, Thabane L, et al. CONSORT 2010 statement: extension to randomised pilot and feasibility trials. The BMJ. 2016;355. 10.1136/bmj.i5239 PMC5076380 27777223

[54] Billingham SAM, Whitehead AL, Julious SA (2013). An audit of sample sizes for pilot and feasibility trials being undertaken in the United Kingdom registered in the United Kingdom Clinical Research Network database. BMC Med Res Methodol..

[55] Hertzog MA (2008). Considerations in determining sample size for pilot studies. Res Nurs Health..

[56] Whitehead AL, Julious SA, Cooper CL, Campbell MJ (2016). Estimating the sample size for a pilot randomised trial to minimise the overall trial sample size for the external pilot and main trial for a continuous outcome variable. Stat Methods Med Res..

[57] Sealed Envelope Ltd. Create a blocked randomisation list [Internet]. Sealed Envel. 2017 [cited 2017 Aug 28]. Available from: https://www.sealedenvelope.com/simple-randomiser/v1/lists.

[58] Craig P, Dieppe P, Macintyre S, Michie S, Nazareth I, Petticrew M (2013). Developing and evaluating complex interventions: the new Medical Research Council guidance. Int J Nurs Stud..

[59] Stringer E (2007). Action Research.

[60] Rycroft-Malone J, Seers K, Titchen A, Harvey G, Kitson A, McCormack B (2004). What counts as evidence in evidence-based practice?. J Adv Nurs..

[61] Sidani S, Braden CJ (2011). Design, evaluation, and translation of nursing interventions.

[62] Michie S, van Stralen MM, West R (2011). The behaviour change wheel: a new method for characterising and designing behaviour change interventions. Implement Sci..

[63] Michie S, Richardson M, Johnston M, Abraham C, Francis J, Hardeman W (2013). The behavior change technique taxonomy (v1) of 93 hierarchically clustered techniques: building an international consensus for the reporting of behavior change interventions. Ann Behav Med Publ Soc Behav Med..

[64] Knafl K, Deatrick J, Gallo A, Holcombe G, Bakitas M, Dixon J (2007). The analysis and interpretation of cognitive interviews for instrument development. Res Nurs Health..

[65] Francisco VT, Butterfoss FD (2007). Social validation of goals, procedures, and effects in public health. Health Promot Pract..

[66] Thabane L, Ma J, Chu R, Cheng J, Ismaila A, Rios LP (2010). A tutorial on pilot studies: the what, why and how. BMC Med Res Methodol..

[67] Keogh-Brown MR, Bachmann MO, Shepstone L, Hewitt C, Howe A, Ramsay CR (2007). Contamination in trials of educational interventions. Health Technol Assess Winch Engl.

[68] Lingler JH, Schmidt K, Gentry A, Hu L, Terhorst L (2014). Perceived Research Burden Assessment (PeRBA): instrument development and psychometric evaluation. J Empir Res Hum Res Ethics JERHRE..

[69] Donabedian A (1988). The quality of care: how can it be assessed?. JAMA..

[70] Hughes R. Vignettes. In: Given L, editor. SAGE Encycl Qual Res Methods [Internet]. 2455 Teller Road, Thousand Oaks California 91320 United States: SAGE Publications, Inc.; 2008 [cited 2020 Apr 20]. p. 918–20. Available from: http://methods.sagepub.com/reference/sage-encyc-qualitative-research-methods/n482.xml.

[71] Hughes R, Huby M (2002). The application of vignettes in social and nursing research. J Adv Nurs..

[72] Xu J, Arruda S, Gallo JJ, Wenzel J, Nolan MT, Flowers D (2018). Using vignettes to understand heart failure self-care. J Clin Nurs..

[73] Hughes R, Huby M (2004). The construction and interpretation of vignettes in social research. Soc Work Soc Sci Rev..

[74] McNeil DW, Quentin LL. Motivation in clinical interventions. Int Encycl Soc Behav Sci. 2nd ed. Oxford: Elsevier; 2015. p. 907–913.

[75] Riegel B, Moser DK, Buck HG, Dickson VV, Dunbar SB, Lee CS (2017). Self-Care for the prevention and management of cardiovascular disease and stroke: a scientific statement for healthcare professionals from the American Heart Association. J Am Heart Assoc..

[76] Huygens MWJ, Swinkels ICS, de Jong JD, Heijmans MJWM, Friele RD, van Schayck OCP, et al. Self-monitoring of health data by patients with a chronic disease: does disease controllability matter? BMC Fam Pract. 2017;18. 10.1186/s12875-017-0615-3 PMC5360032 28320330

[77] Thoma A, Farrokhyar F, McKnight L, Bhandari M (2010). How to optimize patient recruitment. Can J Surg..

[78] Brintnall-Karabelas J, Sung S, Cadman ME, Squires C, Whorton K, Pao M (2011). Improving recruitment in clinical trials: why eligible participants decline. J Empir Res Hum Res Ethics JERHRE..

[79] McCann SK, Campbell MK, Entwistle VA (2010). Reasons for participating in randomised controlled trials: conditional altruism and considerations for self. Trials..

[80] Koller A, Miaskowski C, De Geest S, Opitz O, Spichiger E (2013). Supporting self-management of pain in cancer patients: methods and lessons learned from a randomized controlled pilot study. Eur J Oncol Nurs Off J Eur Oncol Nurs Soc..

[81] Preston NJ, Farquhar MC, Walshe CE, Stevinson C, Ewing G, Calman LA, et al. Strategies designed to help healthcare professionals to recruit participants to research studies. Cochrane Methodology Review Group, editor. Cochrane Database Syst Rev. 2016. 10.1002/14651858.MR000036.pub2 PMC8190980 35658160

[82] Redman BK (2007). Responsibility for control; ethics of patient preparation for self-management of chronic disease. Bioethics..

[83] Olivier J, May WL, Bell ML (2017). Relative effect sizes for measures of risk. Commun Stat - Theory Methods..

[84] Andrade C. Understanding relative risk, odds ratio, and related terms: as simple as it can get. J Clin Psychiatry. 2015:e857–61. 10.4088/JCP.15f10150 26231012

[85] El-Masri MM. Terminology 101: number needed to treat in RCTs [Internet]. Can. Nurse. 2015 [cited 2018 Feb 18]. Available from: https://canadian-nurse.com/articles/issues/2015/june-2015/terminology-101-number-needed-to-treat-in-rcts. 26263609

[86] Portney LG, Watkins MP (2015). Foundations of clinical research: applications to practice.

[87] Moore CG, Carter RE, Nietert PJ, Stewart PW (2011). Recommendations for planning pilot studies in clinical and translational research. Clin Transl Sci.

[88] Polit DF, Beck CT (2012). Nursing research: generating and assessing evidence for nursing practice.

[89] Whitehead AL, Sully BGO, Campbell MJ (2014). Pilot and feasibility studies: is there a difference from each other and from a randomised controlled trial?. Contemp Clin Trials..

[90] Heale R, Twycross A (2015). Validity and reliability in quantitative studies. Evid Based Nurs..

[91] Grove SK, Gray JR, Burns N (2015). Understanding nursing research: building an evidence-based practice.

[92] Köberich S (2016). Fostering self-care behaviours through symptom diary use? An exploratory, cross-sectional study about the use of and attitude towards a symptom diary of patients with heart failure. J Res Nurs..

[93] Evans SC, Roberts MC, Keeley JW, Blossom JB, Amaro CM, Garcia AM (2015). Vignette methodologies for studying clinicians’ decision-making: validity, utility, and application in ICD-11 field studies. Int J Clin Health Psychol..

[94] Page SJ, Persch AC (2013). Recruitment, retention, and blinding in clinical trials. Am J Occup Ther..

[95] Hardcastle SJ, Hancox J, Hattar A, Maxwell-Smith C, Thøgersen-Ntoumani C, Hagger MS. Motivating the unmotivated: how can health behavior be changed in those unwilling to change? Front Psychol. 2015;6. 10.3389/fpsyg.2015.00835 PMC4468355 26136716

[96] Sidani S, Braden CJ. Selecting, training, and addressing the influence of interventionists. Des Eval Transl Nurs Interv. 1st ed. Chichester: John Wiley & Sons; 2011. p. 111–124.

[97] World Medical Association (2013). World medical association declaration of helsinki: ethical principles for medical research involving human subjects. JAMA..

[98] National Commission for the Protection of Human Subjects of Biomedical and Behavioral Research (1978). The Belmont report: ethical principles and guidelines for the protection of human subjects of research.

[99] German Clinical Trials Register. German Clinical Trials Register (DRKS) [Internet]. Ger. Clin. Trials Regist. 2019 [cited 2019 Jun 12]. Available from: https://www.drks.de/.

